# Microbial Communication, Cooperation and Cheating: Quorum Sensing Drives the Evolution of Cooperation in Bacteria

**DOI:** 10.1371/journal.pone.0006655

**Published:** 2009-08-17

**Authors:** Tamás Czárán, Rolf F. Hoekstra

**Affiliations:** 1 Ecology and Theoretical Biology Research Group of the Hungarian Academy of Science and Eötvös University, Budapest, Hungary; 2 Laboratory of Genetics, Wageningen University, Wageningen, The Netherlands; University of Hyderabad, India

## Abstract

An increasing body of empirical evidence suggests that cooperation among clone-mates is common in bacteria. Bacterial cooperation may take the form of the excretion of “public goods”: exoproducts such as virulence factors, exoenzymes or components of the matrix in biofilms, to yield significant benefit for individuals joining in the common effort of producing them. Supposedly in order to spare unnecessary costs when the population is too sparse to supply the sufficient exoproduct level, many bacteria have evolved a simple chemical communication system called quorum sensing (QS), to “measure” the population density of clone-mates in their close neighborhood. Cooperation genes are expressed only above a threshold rate of QS signal molecule re-capture, i.e., above the local quorum of cooperators. The cooperative population is exposed to exploitation by cheaters, i.e., mutants who contribute less or nil to the effort but fully enjoy the benefits of cooperation. The communication system is also vulnerable to a different type of cheaters (“Liars”) who may produce the QS signal but not the exoproduct, thus ruining the reliability of the signal. Since there is no reason to assume that such cheaters cannot evolve and invade the populations of honestly signaling cooperators, the empirical fact of the existence of both bacterial cooperation and the associated QS communication system seems puzzling. Using a stochastic cellular automaton approach and allowing mutations in an initially non-cooperating, non-communicating strain we show that both cooperation and the associated communication system can evolve, spread and remain persistent. The QS genes help cooperative behavior to invade the population, and *vice versa*; cooperation and communication might have evolved synergistically in bacteria. Moreover, in good agreement with the empirical data recently available, this synergism opens up a remarkably rich repertoire of social interactions in which cheating and exploitation are commonplace.

## Introduction

Cooperation – behavior that benefits other individuals – is not easy to explain from an evolutionary perspective, because of its potential vulnerability to selfish cheating. A classic example is formed by the so-called tragedy of the commons [1]. A commons pasture is used by many herders, and the best strategy for an individual herder is to add as many cattle as possible, even if this eventually causes degradation of the pasture. The unfortunate outcome follows from the fact that the division of the costs and benefits of adding additional animals is unequal: the individual herder gains all of the advantage, but the disadvantage is shared among all herders using the pasture. Therefore, although cooperation (involving restraint in the input of animals) among the herders would yield the highest benefit for them as a group, each individual herder will be tempted to cheat by adding additional animals, causing the cooperation to break down.

The basis for evolutionary explanations of cooperation is provided by Hamilton's inclusive fitness (kin selection) theory [2]. Individuals gain inclusive fitness through their impact on their own reproduction (direct fitness effects) as well as through their impact on the reproduction of related individuals (indirect fitness effects) (see also [3]). Altruistic cooperative behavior (costly to the actor and beneficial to the recipient) can only be explained by indirect fitness effects. By helping a close relative reproduce, an individual is indirectly passing copies of its genes on to the next generation.

Another theoretical approach considers the evolution of cooperation in terms of two-level selection, namely between and within groups, rather than partitioning individual fitness into direct and indirect components. Cooperation is favored when the response to between-group selection is greater than the response to within-group selection. From yet another perspective, altruism will be favored by natural selection if carriers of altruistic genotypes are sufficiently overcompensated for their altruistic sacrifice by benefits they receive from others. In other words, there should be assortment between altruists and the helping behaviors of others [4]. Perhaps the most likely mechanism for such assortment is ‘population viscosity’ (limited dispersal), causing the offspring of cooperators to remain spatially associated. These different theoretical approaches do not contradict each other but emphasize different aspects of altruistic behavior [5], [6], [4].

Although most studies of cooperation have been done on animals, there is a fast growing new field of socio-microbiology studying cooperative behaviors performed by microorganisms [7], [8], [9], [10]. Consider a population of bacteria, in which individual cells are producing some public good. Public goods are costly to produce and provide a benefit to all the individuals in the local group. Examples of public goods are exoproducts like virulence factors damaging the host, enzymes for the digestion of food sources, surfactants for facilitating movement, and nutrient scavenging molecules such as siderophores. In many instances microbial cooperation is regulated by quorum sensing.

Quorum sensing (QS) involves the secretion by individual cells of ‘signaling’ molecules. When the local concentration of these molecules has reached a threshold, the cells respond by switching on particular genes. In this way individual cells can sense the local density of bacteria, so that the population as a whole can make a coordinated response. In many situations bacterial activities, such as the production of the mentioned public goods, are only worthwhile as a joint activity by a sufficient number of collaborators. Regulation by QS would allow the cells to express appropriate behavior only when it is effective, thus saving resources under low density conditions. Therefore, QS has been interpreted as a bacterial communication system to coordinate behaviors at the population level [11], [12]. However, its evolutionary stability is somewhat problematic, since cooperative communication is vulnerable to cheating. For example, a signal-negative (mute) strain does not have to pay the metabolic cost of signal production, and a signal-blind (deaf) strain does not pay the cost of responding. Both type of mutants may still benefit from public goods produced in their neighborhood and have actually been observed among environmental and clinical isolates [13], [14] The question then is, under what conditions cheating strains will increase to such an extent that QS breaks down as a regulatory system of cooperative behavior – perhaps with the consequence that the cooperative behavior itself cannot be maintained.

Brookfield [15] and Brown & Johnstone [16] have analysed models of the evolution of bacterial quorum sensing. Although differing in modelling approach, both have studied the evolution of QS in the context of explicit 2-level selection, where selection at the indidual level operates against cooperation, while selection at the group level favors QS. These studies conclude that under fairly broad conditions either stable polymorphism may arise in bacterial populations between strains that exhibit QS and strains that do not [15] or the average resource investment into quorum signalling takes positive values, the actual investment depending on group size and within-group relatedness [16]. Since kin selection appears to be central for the evolution of altruistic cooperation, it is required that cooperation preferentially takes place among related individuals. As Hamilton [2] suggested, this could be brought about either by kin discrimination or by limited dispersal. The first mechanism may play some role in microbial communities, for example if a public good produced by a specific strain can only be utilized by clonemates [10]. However, limited dispersal is probably much more important in microbes because due to the clonal reproduction mode it would tend to keep close relatives together. This implies that the spatial population structure plays a key role in the evolution of bacterial cooperation.

In a previous work [17] we have analyzed the evolutionary stability of QS using a cellular automaton approach, which is eminently suitable to investigate the role of spatial population structure. There we asked whether QS could be stable as a regulatory mechanism of bacteriocin (anti-competitor toxin) production, and concluded that it could be maintained only when the competing strains were unrelated, and not when the bacteriocin is aimed at related strains which can share the signaling and responding genes involved in QS.

Here, we analyze a much more general model of the evolution of QS regulated cooperation, again using the cellular automaton (CA) approach. In fact, QS regulated cooperation can be viewed as a superposition and interaction between two cooperative behaviors: the cooperative QS communication system which coordinates another cooperative behavior (e.g. production of a public good). Both forms of cooperation are potentially vulnerable to being parasitized by cheating strains. We allow the reward and the cost of cooperation, the level of dispersal and the sensitivity of the QS system (the signal strength required to induce production of a public good) to vary, and ask for which parameter combinations cooperation and QS will evolve and be maintained, to what extent the presence of a QS system affects the evolution and maintenance of cooperation, how vulnerable the system is for social cheating and how equilibrium levels of QS and cooperation depend on the parameter values.

## Methods

The model we use is a two-dimensional cellular automaton (CA) of toroidal lattice topology. Each of the 300×300 grid-points of the square lattice represent a site for a single bacterium; all the sites are always occupied, i.e., bacteria may replace each other, but may not leave empty sites. The inhabitants of the sites may differ at 3 genetic loci: locus **C** for cooperation (production of a public good), the other two for quorum sensing: locus **S** for producing the signal molecule and locus **R** for signal response, which includes the signal receptor and the signal transduction machinery that triggers the cooperative behaviour when the critical signal concentration has been reached. Each of these loci can harbour either a functional allele denoted by a capital letter (**C**, **S** and **R**), or an inactive allele denoted by a small letter (**c**, **s**, and **r**). Thus the bacteria can have 2^3^ = 8 different genotypes, each paying its own metabolic cost of allele expression on the 3 loci (Table 1) besides the basic metabolic burden *M_0_* that is carried by all individuals.

**Table 1:** The 8 possible genotypes of the cooperation-quorum sensing system and the corresponding total metabolic costs *m_e_* of gene expression.

GENOTYPE	PHENOTYPE	Total cost *m_e_* (with *m_c_* = 30.0)	Total cost *m_e_* (with *m_c_* = 10.0)	
**csr**	“Ignorant”	0.0	0.0	
**csR**	“Voyeur”	1.0	1.0	
**cSr**	“Liar”	3.0	3.0	
**cSR**	“Lame”	4.0	4.0	
**Csr**	“Blunt”	30.0	10.0	
**CsR**	“Shy”	31.0	11.0	
**CSr**	“Vain”	33.0	13.0	
**CSR**	“Honest”	34.0	14.0	

Cooperation can be costly (*m_c_* = 30.0; left column) or relatively cheap (*m_c_* = 10.0; right column). Cost of QS signalling: *m_s_* = 3.0; Cost of QS response: *m_r_* = 1.0.

*Fitness effects of cooperation*: The product of the cooperating **C** allele is supposed to be an excreted ‘public good’ molecule such as an exo-enzyme for extracellular food digestion. It may increase the fitness of a bacterium, provided there are at least *n_q_* bacteria (possibly, but not necessarily, including itself) expressing the **C** allele as well within its 3×3-cell neighbourhood; *n_q_* is the quorum threshold of cooperation. An individual can only obtain a fitness benefit from cooperative behaviour in its neighbourhood if at least *n_q_* cooperators are present in that neighbourhood. On the other hand, cooperation carries a fitness cost which is always paid by the cooperator whether or not it enjoys the benefits of cooperation. The cost of cooperation is the metabolic burden associated with the production of the public good. That is, cooperation (expressing **C**) carries an inevitable fitness cost and a conditional fitness benefit. We study the effects of a high as well as a low cost of cooperation. Of course for cooperation to be feasible at all the benefit has to outweigh the cost.

### Fitness effects of quorum sensing

Cells carrying genotype**.S.** (for the genotype notation, see Table 1) produce the quorum signal molecule, whereas **R** genotypes will respond to a sufficient amount of signal in their immediate environment. Both the expression of **S** and of **R** imply a fitness cost as well, because producing the signal and running the response machinery takes metabolic resources, although less than cooperation itself [18], [13]. The fitness benefit of a QS system is an indirect one: communication using a signalling system may spare unnecessary costs of futile attempts to cooperate whenever the local density of potential cooperators is lower than the quorum *n_q_*. For this communication benefit to be feasible, the QS machinery altogether has to be much cheaper (in terms of metabolic costs) than cooperation itself, otherwise constitutive (unconditional and permanent) cooperation would be a better option for the bacterium, and resources invested into QS would be wasted. Thus the ordering of the metabolic fitness costs of cooperation and QS are assumed to be 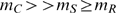. The inactive alleles **c**, **s** and **r** carry no metabolic cost: 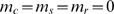


### The effect of the quorum sensing genes on the cooperation gene

The quorum signal is supposed to be the regulator of cooperation: bacteria with a **C.R** genome (i.e., those carrying a functional cooperation allele **C** and a working response module **R**) will actually express the **C** gene (i.e., cooperate) only if there is a sufficient quorum *n_q_* of signallers (**.S.** individuals) within their neighbourhood. That is, **C.R** cells wait for a number of “promises” of cooperation in their 3×3-cell neighbourhood before they switch to cooperating mode (produce the public good) themselves. **C.r** genotypes do not have a functioning response module, therefore they produce the public good constitutively.

### Selection

Individuals compete for sites. Competition is played out between randomly chosen pairs of neighbouring cells, on the basis of the actual net metabolic burdens *M(1)* and *M(2)* they carry. The net metabolic burden *M*(*i*) of an individual *i* is calculated as the sum of the basic metabolic load *M_0_* carried by all individuals and the total metabolic cost *m_e_*(*i*) of the actual gene expressions at the three loci concerned (see Table 1), multiplied by the unit complement of the cooperation reward parameter (1 – *r*) if it is surrounded by a sufficient quorum of cooperators:
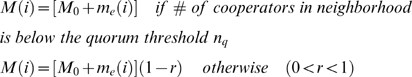



Thus, successful cooperation reduces the total metabolic burden in a multiplicative fashion. The relative fitness of individual *i* is defined as its net metabolic burden relative to the basic metabolic load as 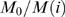. In practice, the outcome of competition is determined by a random draw, with chances of winning weighted in proportion to the relative fitnesses. The winner takes the site of the loser, replacing it by a copy of itself.

### Mutations

During the takeover of a site by the winner of the competition the invading cell, i.e., the copy of the winner occupying the site of the loser, can change one of its 3 alleles (chosen at random) from functional to inactive or *vice versa*. We call these allele changes “mutations”, but in fact they can be due to either mutation or some other process like transformation or even the immigration of individuals carrying the “mutant” allele. The point in allowing allele changes both ways (losing and obtaining them) is to maintain the presence of all six different genes (**C**, **c**, **S**, **s**, **R**, **r**) in the population so that the system doesn't get stuck in any particular genetic state because of the lack of alternative alleles. Thus, each of the six possible allele changes may have a positive probability. Mutations are independent at the three loci – e.g., the quorum signal gene **S** can be lost without losing the response module **R** at the same time; the resulting mutant will be “mute” yet still able to respond to quorum signals.

### Diffusion

Each competition step may be followed by a number (*D*) of diffusion steps. One diffusion step consists of the random choice of a site, and the 90° rotation of the 2×2 subgrid with the randomly chosen site in its upper left corner. Rotation occurs in clockwise or anticlockwise direction with equal probability [19]. *D* is the diffusion parameter of the model: it is proportional to the average number of diffusion steps taken by a cell per each competitive interaction it is engaged in. Larger *D* means faster mixing in the population. Since one diffusion move involves 4 cells, *D* = 1.0 amounts to an expected number of 4 diffusion steps per interaction per cell. In the simulations we use the range 0.0≤*D*≤1.0 of the diffusion parameter, and occasionally much higher values (*D* = 15.0) as well.

### Initial states and output

At *t* = 0 the lattice is “seeded” either by the “Ignorant” (**csr**) genotype on all sites, or the initial state is a random pattern of all the 8 possible genotypes present at equal proportions. We simulate pairwise competitive interactions, mutations and diffusive movements for *N* generations. One generation consists of a number of competition steps equal to the number of sites in the lattice, so that each site is updated once per generation on average. In the majority of simulations we have applied mutation rates of 10^−4^ both ways at each locus, which is equivalent to an average of 9 mutation events per generation within the whole habitat. The three functional alleles have a positive cost of expression, constrained by the relation 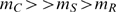 (the actual values used throughout the simulations are given in Table 1).

### Simulations

With the initial conditions specified above we follow the evolution (the change in allele frequencies) for both cooperation and the two components of quorum sensing. We investigate the qualitative or quantitative effects on the evolution of cooperation and quorum sensing of the crucial parameters of the model: the fitness reward of cooperation (*r*), the metabolic cost of cooperation (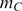), the intensity of diffusive mixing (*D*) and the quorum threshold (*n_q_*). The simulations have been run until the relative frequencies of the three focal alleles (**C**, **S** and **R**) approached their quasi-stationary values. This could be achieved within 10.000 generations in most cases. The first few simulations have been repeated 3 times with each parameter setting, using different random number arrays, but since variation in the results was very small at a lattice size of 300×300 in all cases, and each run took a long time to finish, we stopped producing replicate runs.

During the simulations we record and plot the time series of the 8 different genotype frequencies, from which the frequencies of the three functional alleles can be calculated and plotted against time.

### Evaluation of the model outputs

The simulation results are recorded as 10.000-generation time series of genotype frequencies and spatial patterns of the genotypes. With regard to allele frequencies we asked the following question: are the genes for cooperation (**C**) and quorum sensing (**S** and **R**) selected for beyond their respective mutation-selection equilibria based on the metabolic selection coefficients *s_C_* = (*M_C_ −M_0_*)/*M_C_, s_S_* = (*M_S_ −M_0_*)/*M_S_* and *s_R_* = (*M_R_ −M_0_*)/*M_R_* and the (uniform) mutation rate *μ* ? For example, relative frequencies of the cooperating allele above its mutation-selection equilibrium 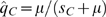 indicate a net fitness benefit of cooperation and thus positive selection for the **C** allele. 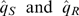 can be calculated the same way.

## Results

### The evolution of cooperation without quorum sensing

We first have performed simulations with the QS functions disabled (mutation rates in both ways set to 0.0 at the **S** and the **R** loci). Without QS allowed, the only possible genotypes are the “Ignorant” (no cooperation) and the “Blunt” (unconditional cooperation), of course.

### (1) cooperation is relatively costly (m_c_ = 30)

**Figure 1:**
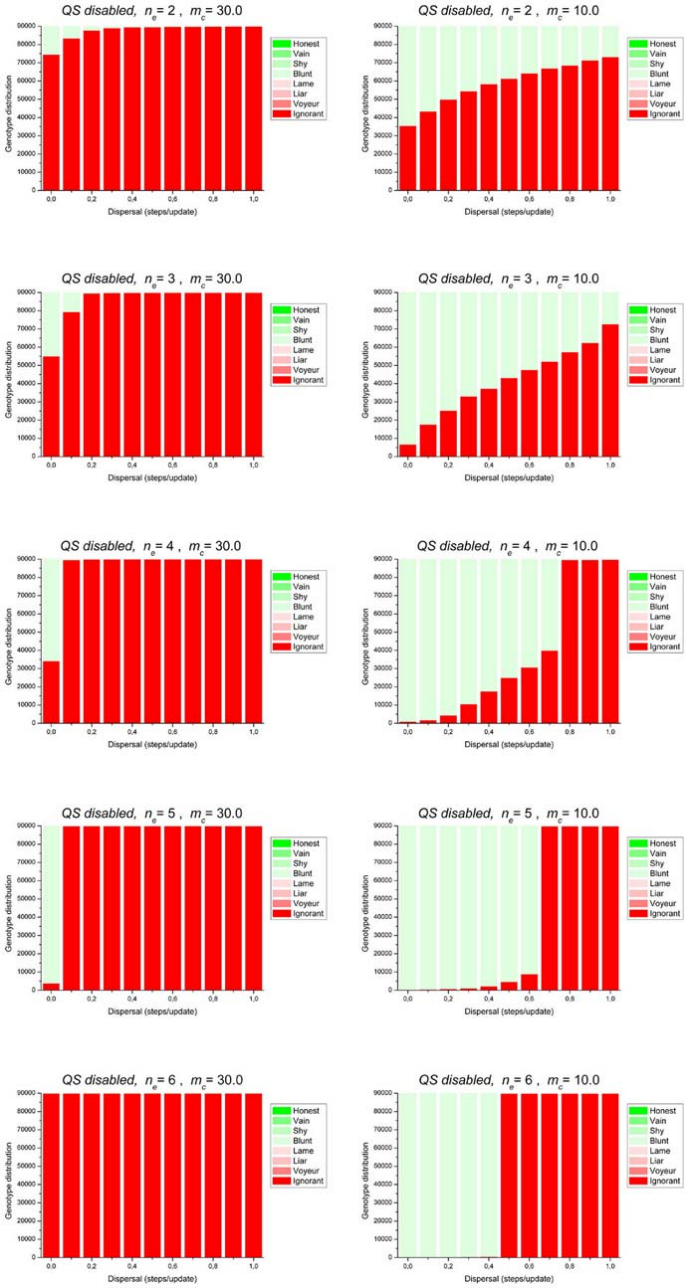
Stationary genotype distributions of the QS-disabled set of simulations. Fixed parameters: basic metabolic burden: *M_0_* = 100.0; metabolic cost of quorum signal production: *m_s_* = 3.0; metabolic cost of quorum signal response: *m_r_* = 1.0; fitness reward factor: *r* = 0.9; mutation rates: *μ_s_* = *μ_r_* = 0.0, *μ_c_* = 10^−4^. Screened parameters: metabolic cost of cooperation (*m_c_*), quorum threshold (*n_e_*) and dispersal (*D*). Simulations lasted for 10.000 generations and they were initiated with the “All-Ignorant” (csr) state.

The left column in Fig. 1 summarizes the results. Cooperation is only selected under a very low degree of dispersal. This confirms the essential role played by kin selection in the evolution of cooperation, since low dispersal in a microbial population implies that most social interactions are among related individuals. With a low quorum threshold (only few cooperators are necessary to provide the benefit to all the immediate neighbors) there is much scope for non-cooperators to parasitize, because sufficiently often they can enjoy the benefit from cooperative neighbors without paying the cost of cooperation themselves; therefore, with n_q_ = 2 and n_q_ = 3, only a minority of the population will consist of cooperator genotypes. With n_q_ = 4 and n_q_ = 5 there is obviously less opportunity for parasitism, and cooperators reach higher frequencies. However, then the system becomes more sensitive to the effects of spatial mixing; with n_q_ = 6 even at D = 0 successfully cooperating neighborhoods are disintegrated more often than that they are formed or maintained, and cooperative behavior is no longer selected.

### (2) cooperation is relatively cheap (m_c_ = 10)

Qualitatively the same trends are apparent when cooperation is less costly (Fig. 1, right column). Cooperation is maintained over a broader range of diffusion rates, compared to the case of costly cooperation. Clearly, with less costly cooperation, occasional futile cooperation attempts (when the number of cooperators in a neighbourhood is less than the quorum) are less deleterious. With increasing quorum threshold the scope for parasitism by non-cooperators becomes smaller and as a consequence a larger fraction of the population will consist of cooperators, as long as neighborhoods sufficiently often contain at least a quorum of cooperators. Above a certain level of population mixing this is no longer the case, and then cooperation does not evolve.

### The evolution of cooperation and quorum sensing

**Figure 2:**
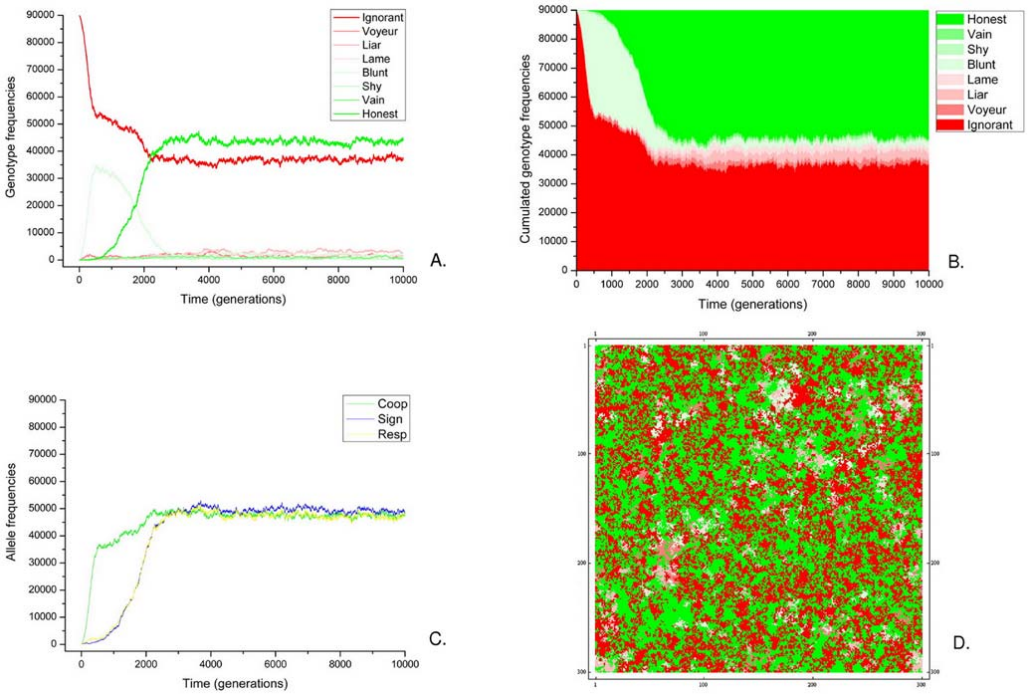
Details of a single QS-enabled simulation. Parameters as in Fig. 1, except for *μ_s_* = *μ_r_* = 10^−4^; *m_c_* = 30.0, *n_e_* = 3 and *D* = 0.0. Time evolution of A.: genotype frequencies; B.: genotype distribution; C.: allele frequencies. D.: The spatial pattern of genotypes at *T* = 10.000.

In the next series of simulations we allow cooperation and quorum sensing to evolve simultaneously, and allowing mutations at all three loci from inactive to functional and *vice versa* with probability μ = 10^−4^. Fig. 2 shows as an example the evolution of the genotype and allele frequencies in a run of the simulation model with a high cost of cooperation and a relatively cheap quorum sensing system (*m_C_* = 30.0, *m_S_* = 3.0, *m_R_* = 1.0), medium quorum threshold (*n_e_* = 3), high cooperation reward (*r* = 0.9) and no diffusion (*D* = 0.0).

The first invading genotype is the “Blunt” one (**Csr**) which cooperates unconditionally but lacks QS. However, as soon as the “Blunt” type reaches a high frequency in the population, the adoption of QS genes obviously becomes profitable, because the “Honest” (**CSR**) genotype takes over, ultimately excluding the “Blunt” one. The “Honest” takeover renders the stationary population essentially dimorphic: the great majority of the individuals are either “Ignorant” or “Honest”. The remaining 6 genotypes are present at very low frequencies, close to their metabolic mutation-selection equilibria. What we end up with is thus the coexistence of cooperating-communicating cells (“Honest”) and parasitic ones (“Ignorant”).

### The effects of changing the quorum threshold and diffusion

#### (1). Cooperation is costly (m_C_ = 30.0)

**Figure 3:**
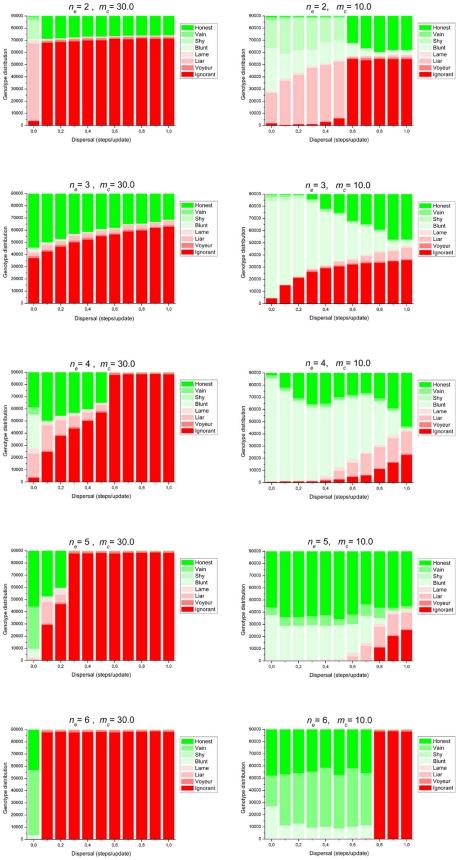
Stationary genotype distributions of the QS-disabled set of simulations. All parameters as in Fig. 1. except *μ_s_* = *μ_r_* = 10^−4^.

Keeping the costs *m_C_*, *m_S_*, *m_R_* and the cooperation reward *r* constant, we have systematically screened the effects of the quorum threshold *n_q_* and the diffusion parameter *D* on the evolution of cooperation and quorum sensing (Fig. 3, left column). Comparison with the corresponding cases without the possibility of QS (Fig. 1, left column) immediately shows that the QS functions of signalling and responding are selected in a large part of the parameter space and that they have a positive effect on the relative frequency of cooperation in the population.

First consider the case of a low quorum threshold (*n_e_* = 2). If the population is not mixed at all (*D* = 0.0), cooperators do not need an intact QS machinery to have a reliable cue on the presence of cooperating neighbors: with a high chance at least one clone mate (mother or daughter) is always around, and that is sufficient for them to enjoy the cooperation reward during their next interaction. This is why the great majority of cooperators have disposed of one or both QS alleles (**S** and **R**) at *D* = 0.0. Most cooperators are of the “Shy” (**CsR**) genotype, which responds to quorum signals and cooperates accordingly, but does not itself produce the signal. Parasites capitalize on this feature by issuing the signal only, thereby persuading the “Shy” type to cooperate. This results in the parasite population to become, to an overwhelming majority, of the “Liar” (**cSr**) type which is the exact complement of the “Shy” one: it possesses the only functional allele that “Shy” is missing. Since the quorum signal is necessary for the onset of cooperation in “Shy” individuals, the interaction between these two dominant genotypes can be interpreted both as parasitism and as a peculiar type of “division of labour”. The latter, less antagonistic component of the interaction immediately disappears with the introduction of the slightest diffusion into the system. At and above *D* = 0.1, the diffusion in the population creates already too many neighborhoods that do not contain the required two **C** and two **S** alleles distributed over separate genotypes (i.e two “Shy” and two “Liar” types), and the presence of **CSR** (“Honest”) is selected, guaranteeing successful cooperation as soon as two such genotypes are present in a neighborhood. This leaves ample space for **csr** (“Ignorant”) parasites of course, which reach high frequencies. This will be true even at fairly high diffusion rates.

The *n_q_* = 3 case has already been described in some detail above (Fig. 2). The special feature of this series of simulations is that the QS system is always adopted by the cooperators, even without diffusion. This might be accounted for by the fact that at about 50–60% (or less) of the population cooperating, the presence of 3 cooperators within a 9-individual neighbourhood is far from guaranteed, making QS well worth its cost for the cooperators. Therefore both QS alleles (**S** and **R**) spread and become established within the cooperating population. Consequently parasites do not need to issue fake quorum signals to access the benefit. Increasing diffusion gradually reduces the likelihood of maintaining three cooperators in a neighbourhood, resulting in a lower level of cooperation in the population. At very intensive diffusion (*D* = 6.0 in this parameter setting) both cooperation and QS disappear together abruptly, and the stage is left for the parasitic “Ignorant” type. Apparently then successful cooperation will be so rare that cooperators are losing more due to the cost of operating the QS machinery, than gaining from the cooperation benefit. Consequently, their relative fitness shrinks below that of the “Ignorant” type, and they vanish.

**Figure 4:**
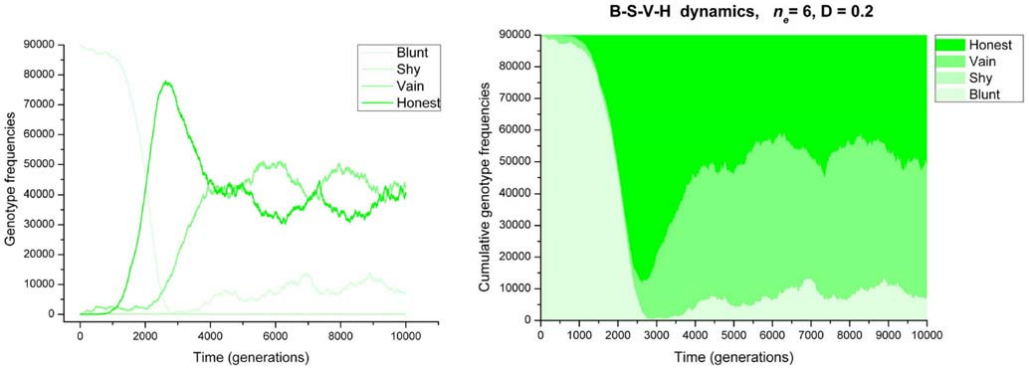
The evolution of QS in a population with the cooperating C allele fixed. Parameters as in Fig. 3, with *n_e_* = 6 and *D* = 0.2. The simulation was started from the “All-Blunt” initial state.

The *n_q_* = 4, 5 and 6 cases are similar to the *n_q_* = 3 case, except for two important aspects. One is that, due to the high quorum threshold, the system becomes more sensitive to spatial mixing. For *n_q_* = 4, the upper limit of the diffusion parameter that still allows the cooperation and QS alleles to persist is *D* = 0.5; for *n_q_* = 5 it is *D* = 0.2 and for *n_q_* = 6 cooperation is only maintained in the absence of spatial mixing (D = 0). Above these *D* values, successfully cooperating clumps (neighbourhoods with *n_q_* or more cooperators) are disintegrated by too intensive mixing, at a rate faster than they are built by interactions. Second, at zero diffusion (*D* = 0.0), for *n_q_* = 4, 5 and 6, cooperators increase in abundance and they tend to lose one or both components of the QS system, unlike in the *n_q_* = 3 simulation. The reason for the loss of the communication device is that cooperators become so common, that QS is no longer needed to find out whether there is a sufficient number of cooperators present in the immediate neighbourhood. Constitutively cooperating genotypes like “Blunt” and “Vain” increase in frequency because they do not pay the (complete) cost of QS. At *n_q_* = 4 the “Honest” type is maintained at about 30%, because QS is still sufficiently often useful, with almost 30% of the population consisting of non-cooperators. Here most of the non-cooperating strains are of the “Liar” type: in neighborhoods with fewer than *n_q_* = 4 “Honest” individuals, their signalling helps to persuade the latter to cooperate. At *n_q_* = 5 and *n_q_* = 6 with zero diffusion, the simulations bring an interesting strategic aspect of QS to the light. Although almost 100% of the population is cooperating, the fully quorum sensing “Honest” type is maintained at some 30–50% of the population. This is at first sight surprising, since the presence in local neighborhoods of a quorum of cooperators is practically guaranteed. However, here QS appears to function as a mechanism to *avoid* expression of the cooperative behavior when already a sufficient number of unconditionally cooperating (“Blunt”) neighbors are producing the public good. Clearly, when less then *n_q_* cells in a neighbourhood are producing the quorum signal molecule, “Honest” types will not cooperate, thus saving the cost of cooperation while frequently enjoying the cooperation benefit thanks to their unconditionally cooperating neighbors. This explains the fairly high frequency of signalling unconditional cooperators (“Vains”). By enhancing the local concentration of the quorum signal they induce “Honest” cells to cooperate, thereby enhancing the likelihood that a quorum of cooperators is reached. Actually, in situations where cooperation is so attractive that the **C** allele is (almost) fixed, the three cooperating types: “Honest”, “Blunt” and “Vain” display a cyclic interaction pattern (Blunt>Vain>Honest>Blunt) reminiscent of the rock-scissors-paper (RSP) game [20], [21]. A population of “Blunt” is invaded by “Honest” because – as explained above – “Honest” parasitizes on the unconditional cooperation by the “Blunts”. Conversely, an “Honest” population is invaded by “Blunt” and by “Vain”, because they do not pay (part of) the costs of the QS machinery, and “Vain” invades a polymorphic (“Honest”, “Blunt”) population, enhancing the likelihood of a quorum of actual cooperators by inducing “Honest” cells. Fig. 4 shows the evolutionary dynamics of such a population with the cooperating **C** allele fixed.

#### (2). Cooperation is relatively cheap (m_C_ = 10.0)

The right column in Fig. 3 shows the simulation results for less costly cooperation. Just as in the case of costly cooperation, QS alleles are selected in a large part of the parameter space and boost the frequency of cooperators in the population (compare with Fig. 1, right column). In particular, QS enables the maintenance of cooperative behavior at higher levels of population mixing. At low quorum thresholds (*n_q_* = 2 and *n_q_* = 3) and a low rate of diffusion, the QS machinery is too expensive because sufficiently often neighborhoods will contain a quorum of unconditional cooperators, and the QS alleles are not selected. When the rate of spatial mixing increases, the predictability of the local population composition goes down, and QS becomes profitable. At higher quorum thresholds (*n_q_*≥4), we see again that if the population (almost) exclusively consists of (potential) cooperators, QS is selected because its machinery allows cells to *avoid* cooperating when the number of unconditionally cooperating neighbors is already equal to or higher than the quorum. The resulting cooperating population consists of a dynamical coexistence of fully quorum sensing (“Honest”) genotypes, unconditionally cooperators (“Blunt” types) and signalling unconditionally cooperators (“Vains”).

### The effect of decreasing the reward of cooperation

At a lower cooperation reward of *r* = 0.5 neither cooperation nor quorum sensing evolves: the population becomes almost completely uniform “Ignorant” within the entire parameter space. This result is somewhat surprising, given that at *r* = 0.5, successful quorum sensing cooperators like the “Honest” genotype should still enjoy a substantial fitness advantage compared to the “Ignorant”. The total metabolic burden of an “Honest” individual after getting the cooperation reward is 133.0 * 0.5 = 66.5, whereas the “Ignorant” carries a burden of 100.0 units, i.e., the cooperator should have a fitness advantage of about 34% over the parasite. It cannot use it to the full, however, because nearby parasites may take the advantage as well without paying the costs, and those parasites which are successful in doing so carry an even lower (50.0 units) metabolic burden. Apparently a minimum threshold measure of fitness reward is necessary for cooperation to become an option. With the quorum threshold fixed at *n_q_* = 3 and diffusion at *D* = 0.0, we looked for the critical value of the fitness reward by increasing *r* from 0.5 to 0.9, and found it to be *r_c_* = 0.8. This means that the kind of exploitable, broadcasted cooperation, such as producing a public good needs to be highly rewarded for it to be worthwhile to adopt, otherwise parasitism prevails and ultimately eradicates cooperation.

## Discussion

Microorganisms display a wide range of social behaviors, such as swarming motility, virulence, biofilm formation, foraging and ‘chemical warfare’ [7], [21], [8], [9], [10]. These social behaviors involve cooperation and communication. Cooperation often takes the form of a coordinated production and excretion of molecules like enzymes, toxins and virulence factors. In bacteria, this cooperative behavior is typically regulated by quorum sensing (QS), a chemical communication system in which cells produce diffusible molecules and can assess the cell density by sensing the local concentration of these signaling molecules [11], [12], [22], [23]. In fact, QS can be viewed itself as a cooperative behavior to optimize other forms of social behavior. An important issue is the evolutionary stability of cooperation because of its potential vulnerability to social cheating: the occurrence and selection of individuals who gain the benefit of cooperation without paying their share of the costs [2], [10]. We studied the evolution of cooperative behavior in bacteria (e.g. production of a public good) and of a QS system which coordinates this cooperative behavior, using cellular automaton (CA) modeling. This approach allows a fairly precise evaluation of the role played by the spatial population structure, because all bacterial interactions are supposed to occur between neighboring cells.

Our results allow three main conclusions, which we discuss in turn.

### 1. Cooperation only evolves under conditions of limited dispersal

The simulations in which we analysed the evolution of cooperation without QS suggest that cooperation can only evolve when the degree of spatial mixing in the population is low, which implies a high relatedness between neighboring cells. Our model thus confirms the importance of the level of relatedness between interacting individuals and the evolutionary stability of cooperation, as first hypothesized by Hamilton (Hamilton 1964), and demonstrated experimentally in microbial populations [24], [25], [26].

The level of exploitation of cooperative behavior by non-cooperating strains is lowest when the required quorum of cooperators is relatively high and the dispersal rate is low (Fig. 1).

### 2. The presence of cooperative strains in a population always selects for QS and cooperation becomes more common as a consequence of QS

The simulations in which we allow the simultaneous evolution of cooperation and QS suggest that whenever the gene for cooperation is selected, also one or both of the communication genes of the QS system are selected. Moreover, the presence of QS (either partial or complete) allows stable levels of cooperation in regions of the explored parameter space where cooperation without QS cannot invade (compare the corresponding columns in Fig. 1 and Fig. 3). Thus a communication system helps to establish stable cooperation. Of course, communication about willingness to cooperate will only be selected if at least part of the population is able to cooperate, so evolution of QS is not expected in a completely non-cooperating population. But it is not self-evident that QS always should enhance the frequency of cooperating strains in the population. Clearly, QS by cooperative strains is selected if the advantage derived from limiting the actual cooperative behavior to when it is most profitable outweighs its costs. In this way QS causes the gene for cooperation to increase. But QS genes may also be selected in non-cooperators, allowing exploitation of cooperative strains and lowering the frequency of cooperation. This applies in particular to “Liar” strains, non-cooperators which signal willingness to cooperate, which may manipulate fully quorum sensing “Honest” strains to cooperate when actually the number of local cooperators falls below the quorum *n_q_*. As a consequence, these “Honest” cells pay the cost of cooperation but cannot enjoy its benefit.

### 3. The communication – cooperation system as modeled in this study displays a remarkably rich and complex pattern of social interactions in which cheating and exploitation play a significant role

QS not only leads to a higher equilibrium frequency of the cooperation gene, but also allows a striking diversity of social interactions. Of the 8 possible genotypes in our model, defined by the presence/absence of the three functional genes for resp. cooperation, signaling and responding, 6 genotypes may reach appreciable equilibrium frequencies, depending on the precise parameter combinations. Only two mutant types play an insignificant role in the system: “Voyeur” which responds to the signal but is unable to signal and cooperate itself, and “Lame”, which is fully quorum sensing (signaling and responding) but cannot cooperate. Inspection of Fig. 3 reveals the possibility of 5 different stable polymorhisms characterized by domination of two genotypes: [Blunt,Ignorant], [Blunt,Honest], [Shy,Liar], [Honest,Ignorant] [Honest,Vain]; 5 polymorphisms with three dominating genotypes: [Honest, Blunt, Ignorant], [Honest, Blunt, Liar], [Honest, Ignorant, Liar], [Honest, Vain, Blunt], [Shy, Liar, Blunt] and one in which four genotypes reach an appreciable frequency: [Honest, Blunt, Liar, Ignorant]. It is important to note that in all cases some degree of social cheating occurs in the form of exploitation or parasitism. Thus our analysis predicts the large-scale occurrence of social cheating in microbial populations.

Two of the polymorphisms mentioned above merit more elaborate discussion.

### The Janus head of QS

In cases where the cooperation gene is (almost) fixed, one might at first sight expect a monomorphic unconditionally cooperating (“Blunt”) population, because Blunt is the cooperator with the lowest metabolic costs, and in a fully cooperating population QS is not needed to obtain information about the potential level of cooperation. However, we find next to “Blunt” appreciable frequencies of fully quorum sensing (“Honest”) and partially quorum sensing (“Vain”) cells. The reason appears to be that here QS is selected because it allows exploitation of Blunt strains, which unconditionally cooperate. As soon as a quorum of Blunts is present, the other cells need not cooperate themselves in order to profit from the cooperation benefit. Adoption of the QS machinery allows them to do precisely this, since in such cases the level of signal is too low to trigger their cooperative behavior. This phenomenon is an unexpected and novel result, showing that QS not only prevents wasting resources when too few potential cooperators are around, but also allows cells to parasitize on unconditionally cooperating neighbors, when a sufficient number of those are present. It may occur at a large scale when the gene for cooperation is (almost) fixed in the population, due to a favorable benefit/cost ratio of the cooperative behavior and the quorum threshold relatively high. As explained more fully in the Results section, 100% cooperating populations seem to evolve in most cases to a [Blunt, Honest, Vain] mixture, characterized by a cyclic interaction pattern (Blunt>Vain>Honest>Blunt) reminiscent of the rock-scissors-paper (RSP) game [20], [21].

### Spiteful behavior

The second polymorphism we want to call attention to is the coexistence of Honest, Liar and Ignorant, which occurs e.g. at n_q_ = 4 and n_q_ = 5 for certain diffusion values. Clearly, the non-cooperative Ignorant and Liar cells exploit the Honest cells which provide cooperation benefits. Here the selective advantage of Liar is at first sight remarkable, since it pays a higher metabolic cost than Ignorant, and can only expect the same share as Ignorant from the cooperation benefit made available by the Honest cells. The only effect of Liar is to sometimes induce Honest cells to cooperate when actually less than the quorum of Honest is present. Nothing is gained, except that in such cases Honest is paying the cooperation cost without getting the benefit. Thus this coexistence is a clear example of spiteful behavior. Liar lowers the relative fitness of Honest, but also pays a fitness penalty (the cost of signaling) itself.

How do our results relate to previous theoretical and empirical work on the evolution of quorum sensing and cooperation? They confirm the basic result from earlier theoretical analyses of the evolution of QS [15], which predicted a stable polymorphism in microbial populations between Qs and non-QS strains. The conclusion of Brown and Johnstone that the highest level of QS signal expression is expected for intermediate levels of relatedness between interacting strains [16] is confirmed in our study only for cases when the cooperation cost is low and the required quorum threshold is also low. Then the benefit of cooperation is relatively easy to obtain, and the QS machinery is too costly to operate. Only when the spatial population mixing becomes more intensive, causing the predictability of the neighborhood composition to go down, QS becomes profitable. The situation is different for costly cooperation and/or a high quorum threshold. Apparently, then QS is profitable even at a very low rate of dispersal (i.e., at high relatedness between interacting cells) because of the lower level of cooperation in the population and/or the greater sensitiveness to increased dispersal.

The available empirical observations on natural occurrence of QS cheats are mainly from work on *Pseudomonas aeruginosa*: [27], [13], [28], [29], [30], [31]. Although these experimental studies cannot yet be informative with respect to the full spectrum of possible mutants and only focus on one or two QS mutants, they report a considerable level of social cheating, which is in agreement with our study.

Finally, we mention an experimental result that may be of relevance with respect to QS evolution but is not included in this model. It is related to the feedback-regulation of QS signal production: “signal deaf” signaler mutants (in our notation:**.Sr** genotypes) are shown to produce an excess of signal molecules compared to signal responsive ones, because in the latter signal production is downregulated by the extracellular concentration of the signal itself, which response-deficient mutants cannot sense [32], [33]. The effect of signal over-expression on the dynamics of QS evolution require further theoretical work.

In conclusion, we predict that the evolution of QS as a communication system regulating cooperative behavior such as the production of a public good has two striking effects. First, it enables the cooperative behavior to attain a higher frequency in the population, and second, it opens up a remarkably rich repertoire of social interactions in which cheating and exploitation are commonplace.
